# Thermal Insulation Performance of Epoxy-Based Intumescent Coatings: Influence of Temperature-Induced Porosity Evolution on Heat Transfer Resistance

**DOI:** 10.3390/polym17111426

**Published:** 2025-05-22

**Authors:** Taher Hafiz, James Covello, Gary E. Wnek, Stephen Hostler, Edrissa Gassama, Yen Wei, Jiujiang Ji

**Affiliations:** 1 Macromolecular Science and Engineering, Case Western Reserve University, Cleveland, OH 44106, USA; tbh25@case.edu (T.H.); jpc106@case.edu (J.C.); mbakoi.gassama@gmail.com (E.G.); 2 Mechanical and Aerospace Engineering, Case Western Reserve University, Cleveland, OH 44106, USA; srh6@case.edu; 3 Department of Chemistry, Tsinghua University, Beijing 100084, China; weiyen@mail.tsinghua.edu.cn

**Keywords:** fire-resistant coating, thermal conductivity of epoxy coating, heat transfer resistance of coating, porosity evolution, intumescent char formation, COMSOL heat transfer simulation, thermal behavior of polymer coating

## Abstract

This study investigated the thermal performance of reduced super intumescent (RSI) coating, focusing on the correlation between porosity evolution and thermal conductivity under elevated temperature conditions. Porosity development was quantified using scanning electron microscopy (SEM) combined with MATLAB-based image analysis, achieving a maximum porosity of 62% after 60 min of exposure. Thermal degradation was characterized using thermogravimetric analysis (TGA), which recorded a mass loss of 35% between 250 °C and 400 °C, capturing the decomposition kinetics and correlating degradation stages with char formation. Fire protection efficiency was evaluated by employing heat flow meter tests (thermal conductivity reduced from 0.15 W/mK to 0.05 W/mK), methane torch experiments (backside temperature increase delayed by up to 50% compared to uncoated steel), and COMSOL-based heat transfer simulations. The results revealed that the RSI coating’s thermal conductivity decreased as its porosity increased, enhancing its insulation effectiveness. Additionally, the formation of a thermally stable char layer at 400 °C significantly reduced heat transfer to the metal substrate by 66%. These findings support the optimization of bio-derived fire-retardant coatings for passive fire protection applications.

## 1. Introduction

The fire safety of materials used in industrial and structural engineering applications is critical [1], primarily because of the risk of catastrophic thermal failure under high temperatures [2,3]. Intumescent coatings (ICs) have emerged as a key passive fire protection strategy [4,5,6] owing to their significant resistance to heat transfer through expansion and char formation under thermal exposure [6,7,8]. However, although these coatings enhance fire resistance [9], their performance depends on porosity evolution and variations in thermal conductivity, which influence their insulating efficiency [10,11,12]. Understanding and optimizing these relationships is crucial for improving fire-resistant materials and developing predictive models to simulate their behavior in real-world fire scenarios [12]. Recent research highlights the complex interaction between porosity [13] and heat transfer within ICs [14], emphasizing that higher porosity can improve thermal insulation by decreasing heat conduction paths [15], although it may also compromise mechanical properties [16]. Alternative methods, such as anodizing [17] and microarc oxidation in molten salts [3], offer distinct approaches to producing heat-resistant coatings. Anodizing enhances surface oxide layers for improved corrosion and heat resistance but is limited by thin coating thickness (typically <50 μm) and high energy consumption, lacking the expansive char formation characteristic of ICs. Microarc oxidation in molten salts provides thicker, ceramic-like coatings with excellent thermal stability but involves complex processing and higher costs compared to the scalable, cost-effective intumescent approach used here. Studies using scanning electron microscopy (SEM) [6] and thermogravimetric analysis (TGA) have provided critical insights into IC expansion, decomposition, and char formation during fire exposure [1,18,19]. However, a systematic quantification of the influence of porosity on thermal conductivity remains lacking, particularly in coatings designed for structural steel protection under prolonged thermal loads [20,21]. This study investigated the thermal insulation behavior of a reduced super intumescent (RSI) coating under 400 °C exposure, correlating increased porosity (up to 62%) with reductions in thermal conductivity (from 0.15 W/mK to 0.05 W/mK). Using a novel integrated experimental and numerical approach—combining SEM-based porosity quantification with MATLAB-based image processing, TGA mass loss analysis, and COMSOL Multiphysics 6.1 Multiphysics simulations—we provide a comprehensive thermal characterization. This approach advances existing methods by linking microscale porosity dynamics to macroscale fire resistance, offering a new framework for optimizing ICs with enhanced thermal insulation and structural integrity, particularly for oil and gas applications.

### 1.1. Intumescent Coatings and Fire Protection: Motivation and Research Gaps

Passive fire protection systems rely on materials that resist high temperatures and delay heat propagation [22,23,24]. ICs function by expanding and forming an insulating char that decreases heat transfer to the substrate [25]. However, the degree of expansion and porosity formation determines overall thermal performance [26] because the presence of voids and air pockets alters the coating’s effective thermal conductivity (${Κ}_{eff}$) [27,28,29]. Traditional studies on fire-retardant coatings have focused primarily on qualitative assessment of char formation rather than on quantitative analysis of how porosity affects thermal insulation efficiency [30]. Recent advances in computational modeling have enabled a more rigorous evaluation of heat transfer within these expanding layers [31,32,33]. The numerical–experimental integration used in this study offers a novel method for quantifying porosity evolution, thereby bridging the gap between experimental observations and computational predictions of heat transfer behavior.

Recent research on bio-fiber-reinforced polyurethane coatings for high-performance concrete demonstrates the benefits of natural additives in improving structural adhesion and thermal resistance, as well as contributing to sustainability objectives [34]. Building upon these principles, our research explores the synergistic effect of tannic acid and ammonium polyphosphate in forming a stable char structure with enhanced insulation efficiency under thermal exposure.

To ensure industrial relevance, we consulted with industry professionals— a chemist and a polymer materials expert—working in polymer R&D and oil and gas applications. This expert feedback highlighted that technical and regulatory limitations often delay the adoption of new coatings despite promising laboratory results and emphasized the significant impact of porosity on thermal conductivity and coating performance. Additional expert insights emphasized the need for reliable performance data, validated modeling frameworks, and enhanced testing standards to accelerate the adoption of fire-retardant coatings in critical infrastructure. These expert interviews helped shape the direction of this study, to ensure that it met both scientific goals and real industrial needs.

### 1.2. Numerical and Experimental Analysis: Need for Integrated Studies

The effectiveness of ICs is evaluated with fire resistance tests, yet real-world applications require predictive numerical models capable of simulating heat transfer behavior across expanding porous structures [35,36]. Studies, such as those by [27], have developed 1D numerical models for heat conduction in swelling polymers but have lacked comprehensive inclusion of porosity-dependent thermal resistance. Similarly, 2D and 3D [37,38] simulations provide more realistic fire scenarios but require validated experimental data to ensure accuracy [39]. By using MATLAB-based image segmentation for porosity quantification and integrating COMSOL-based thermal modeling, this study presents a multi-scale approach linking microscale porosity variations to macroscale fire resistance performance. High-resolution SEM imaging enabled precise structural characterization, whereas TGA mass loss analysis provided insights into thermal degradation behavior [40,41,42]. Our findings contribute to a scientific framework for optimizing ICs by adjusting porosity parameters to enhance thermal resistance.

### 1.3. Heat Transfer Mechanisms in Intumescent Coatings

Heat transfer in ICs is governed primarily by conduction, convection, and radiation. Conduction occurs within the solid coating layer because of thermal gradients, wherein the expansion of the coating enhances its resistance to heat flow. Expanded coating formations improve fire resistance by decreasing conductive heat transfer [19]. Convection occurs in the porous regions of the expanding coating, where trapped air pockets provide thermal insulation. The extent of convection depends on the coating’s porosity: higher porosity improves insulation but potentially weakens structural integrity [20]. Radiation becomes the dominant heat transfer mode at elevated temperatures, particularly in the outer char layers. The ability of the char to absorb and reflect radiative heat significantly affects its thermal shielding performance [12]. The effectiveness of radiative heat transfer resistance is influenced by the emissivity and composition of the char, which determine its ability to withstand high-intensity thermal exposure [21].

### 1.4. Influence of Porosity on Thermal Insulation

The thermal efficiency of ICs depends on their expansion and void formation during fire exposure [17]. As coatings expand, a porous char structure develops and alters heat transfer by trapping gas pockets that impede conductive and convective heat flow. However, excessive voids may weaken structural integrity and decrease long-term fire resistance [22]. The ${Κ}_{eff}$ is directly influenced by porosity and pore size: an optimal balance between these parameters enhances insulation without compromising mechanical stability [23]. Herein, the effect of the pore size was investigated.

### 1.5. Research Significance and Objectives

By systematically linking porosity, thermal conductivity, and heat transfer resistance, this research provides novel insights into the thermo-physical mechanisms governing ICs. The key objectives of this study were as follows:

Quantify porosity variations in RSI coating using SEM imaging and MATLAB-based image analysis (maximum porosity of 62% at 60 min);

Correlate porosity evolution with changes in under various fire exposure conditions (thermal conductivity reduced from 0.15 W/mK to 0.05 W/mK);

Validate a numerical heat transfer model incorporating porosity-dependent thermal resistance in ICs using COMSOL Multiphysics.

By achieving these objectives, this study enhances scientific understanding of fire-resistant coatings. The findings have implications for next-generation thermal insulation materials in fire protection engineering, offering a novel integration of microscale porosity analysis with macroscale thermal performance for oil and gas applications. This multi-scale approach, validated using precise experimental data and advanced simulations, distinguishes our work from previous studies by providing a robust framework for optimizing ICs with enhanced thermal insulation and structural integrity.

## 2. Experimental Methods

### 2.1. Sample Preparation

In the RSI coating formulation, EPON 828 epoxy resin obtained from Hexion Inc. (Columbus, OH, USA) served as the primary binding agent for high adhesion and chemical resistance. This diglycidyl ether of bisphenol A (DGEBA) resin offers high mechanical strength and thermal stability.

The polyamine (PA)-based hardener (curing agent) Ancamide 903MAV, obtained from Evonik (Essen, Germany), has high crosslinking efficiency and structural stability, and enhances char retention under fire exposure. The interaction between epoxy and PA improves fire resistance and coating integrity in the RSI coating. The char-forming agent, carbonaceous tannic acid (TA) obtained from Sigma-Aldrich (St. Louis, MO, USA), enhanced early char formation, thereby generating a dense, thermally stable protective layer during fire exposure and improving thermal degradation resistance. The phosphorus-based acid source, ammonium polyphosphate (APP; Exolit AP 422 from Clariant Corporation, Muttenz, Switzerland), served as a flame-retardant additive for RSI coating. This compound promoted intumescence through the controlled release of phosphoric acid, thereby enabling the formation of a highly insulating, thermally stable char structure, decreasing heat transfer, and enhancing fire resistance.

The substrate material (Steel Specimens) comprised hot-rolled carbon steel (HRCS) plates purchased from MetalDopt (Cleveland, OH, USA), which were used for testing the RSI coating. The HRCS plates were not pre-treated, to ensure coating adhesion and performance evaluation under realistic industrial conditions. These steel plates represented common structural materials in fire protection applications.

#### RSI Coating Preparation Method for Fire Testing and Specific Concentration

The RSI coating formulation consisted of epoxy-based (EPON 828) resin, PA curing agent (Ancamide 500), APP, and TA. The dry components (TA and APP) were mixed, and PA was subsequently added to the resin binder and gradually added to the dry components with a mechanical vortex mixer to achieve a viscous mixture. To ensure optimal performance, specific concentrations were carefully measured and applied.

The epoxy was mixed with 28 wt% additives, followed by 21.2 wt% PA, stirred for 5–10 min, cast, degassed, and cured at 60 °C for 24 h. Samples were then sanded to a uniform 1 mm thickness for testing. A viscous mixture was evenly applied to an HRCS sheet measuring 127 mm by 6 mm and cured at 60 °C for 24 h. The formulation details are summarized in Table 1 below.

The RSI coating consisted of 65 wt% epoxy resin and 35 wt% additives (TA, APP, and PA) in a 1:3:1 ratio for a 1 mm thickness. The final dry film thickness was verified to maintain consistency across all tested samples.

### 2.2. Fire Testing and Thermal Behavior Assessment

Two experimental fire tests were conducted to evaluate the thermal behavior and heat transfer resistance of the RSI coating applied to steel substrates. The same batch of prepared samples was used in both experiments to ensure consistency in material composition and coating thickness.

#### 2.2.1. Test 1: Diffusive Methane Flame Exposure (Fire Testing and Thermal Behavior Assessment)

**Figure 1:**
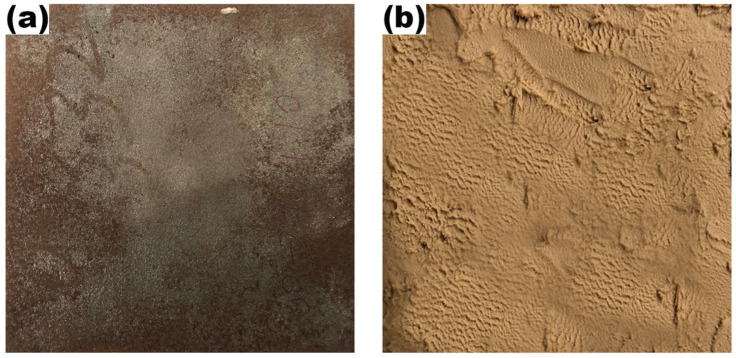
Surface Characteristics of (**a**) steel base and (**b**) its intumescent coating.

The fire testing procedure involved exposure of a square metal specimen (127 mm × 127 mm × 6 mm) to a diffusive methane flame for assessment of the thermal performance of the RSI coating. Figure 1 shows the HRCS substrate before and after coating application.

**Figure 2:**
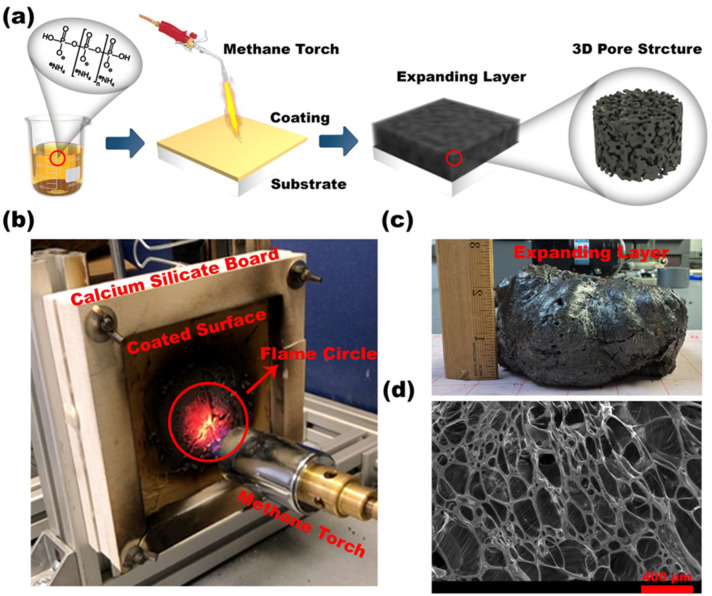
(**a**) Schematic of RSI coating exposure to flame and 3D pore formation; (**b**) real setup for methane torch flame test; (**c**) expanded char layer photograph; and (**d**) SEM of pore structure.

**Figure 3:**
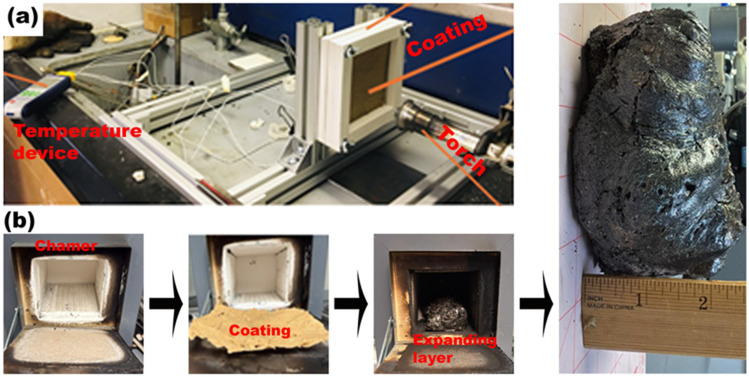
(**a**) Diffusive methane flame exposure; (**b**) thermal stability and char formation of RSI coating under fire exposure.

The coated specimen was mounted in a controlled test setup (custom-built rig, Case Western Reserve University, Cleveland, OH, USA) to replicate realistic fire conditions. As depicted in Figure 2a and Figure 3a, a methane-diffusive torch (Bernzomatic, Columbus, OH, USA) served as the heat source, a temperature measurement device (Omega Engineering, Norwalk, CT, USA) recorded real-time thermal responses, ensuring accurate heat transfer resistance evaluation. The four thermocouples (Omega Engineering, Norwalk, CT, USA) were located in a square grid on the backside, with a vertical and horizontal spacing of 31 mm between them. The test setup also included a calcium silicate backing (Promat, Willebroek, Belgium) to minimize heat loss and focus the flame on the RSI-coated sample. Figure 2a illustrates the schematic of the experimental procedure, starting from bio-derived coating preparation and its direct flame exposure using the methane torch. The coating undergoes a rapid thermal reaction, expanding to form a protective char with a porous 3D microstructure. Figure 2b shows the experimental rig during the flame test, where the flame directly impinges on the coated surface, confined within a defined flame circle. Figure 2c captures the resulting expanding layer, showcasing the swollen char that develops due to gas evolution and intumescence. Finally, Figure 2d presents a scanning electron microscope (SEM) image of the post-exposure char structure, revealing an interconnected pore network that plays a vital role in thermal insulation by trapping hot gases and reducing conductive heat transfer to the substrate. This sequence effectively demonstrates the coating’s transformation under extreme thermal stress and highlights the critical role of microstructural changes, particularly pore formation, in achieving passive fire protection. The consistent pore structure shown in the SEM confirms the material’s ability to form a stable, low-conductivity barrier, making the RSI coating a promising candidate for real-world fire-retardant applications in industrial environments.

**Figure 4:**
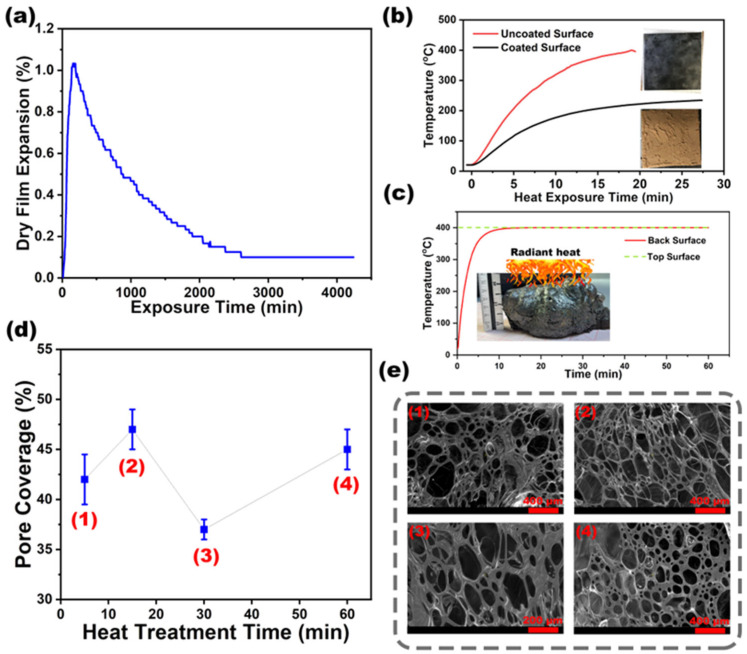
(**a**) Time-dependent expansion behavior of RSI coating under thermal exposure; (**b**) impact of passive fire protection on substrate temperature under direct flame exposure; (**c**) intumescent coating efficiency: delaying heat penetration to steel substrate; (**d**) porosity variation during heat exposure; (**e**) microstructural integrity of expanded RSI coating: voids, matrix, and fire-induced char morphology.

The results from test 1 (Figure 4b) demonstrated the thermal resistance of the RSI-coated surface compared with an uncoated substrate. The temperature–time profile highlighted the effectiveness of the RSI coating in decreasing heat transfer. The uncoated substrate exhibited a faster temperature rise, whereas the protected substrate maintained significantly lower surface temperatures throughout the exposure period.

These findings validated the insulating performance of RSI coatings under direct flame conditions, thus supporting the suitability of these coatings for fire protection applications in steel structures. After the methane flame exposure test, test 2 was conducted to analyze the heat transfer mechanisms under controlled heating conditions.

#### 2.2.2. Test 2: Oven Controlled Heating Experiment (Thermal Heat Chamber)

To further investigate the heat transfer mechanisms of the RSI coating, we subjected the same coated sample to a uniform 400 °C environment inside a thermal heat chamber. This controlled heating condition systematically evaluated char expansion, structural integrity, and thermal resistance without direct flame interaction. Figure 3b shows the sequential stages of the fire resistance behavior, from the intact solid matrix before heating to the formation of an expanded, porous char layer.

To gain deeper insight into the expanded char’s microstructural characteristics, we extracted samples from test 2 and analyzed them with SEM to evaluate porosity distribution, pore size variation, and char morphology, which are critical to thermal insulation efficiency. The RSI coating transitioned from its initial solid state before exposure to an expanded porous char layer after heating (Figure 3b).

The results of test 2 demonstrated the formation of an expanded, porous char layer, which plays a critical role in the thermal resistance and structural stability of RSI coatings. Although the experimental fire tests provided insights into the macroscopic heat transfer behavior, further characterization is necessary to understand the developed char’s microstructural properties, thermal stability, and expansion mechanisms. Therefore, we next conducted advanced characterization techniques to achieve a comprehensive evaluation. The following section focuses on the thermal, microstructural, and expansion properties of the RSI coating, as determined through analytical methods, including bench-scale pyrolysis, thermal conductivity measurements, and thermal decomposition analysis.

### 2.3. Characterization of RSI Coatings: Thermal, Microstructural, and Expansion Analysis

#### 2.3.1. Bench Scale Pyrolysis

Bench-scale pyrolysis was used to evaluate the expansion behavior of RSI coatings under controlled burning conditions with a methane burner. The expansion test measured the RSI formulation’s real-time intumescence and provided insights into char stability, expansion kinetics, and gas release dynamics. The RSI coatings exhibited intumescent behavior, expanding under thermal exposure (Figure 4a). The peak dry film expansion was approximately 1.2% within the first few minutes. This expansion was driven by decomposing intumescent additives (APP and TA), which released gases that induced swelling and formed an insulating char layer. However, the resulting char showed low mechanical resistance and efficiently detached under light agitation of the substrate. Over time, the expansion gradually declined, thus indicating char stabilization and densification.

The reaction neared completion after 30 min and plateaued after 50 min, thus suggesting the formation of a thermally stable, protective char layer. Compared with other ICs, the RSI coatings exhibited lower expansion but stronger adhesion suggestive of a more compact and stable char structure.

#### 2.3.2. Thermal Conductivity Measurement

**Figure 5:**
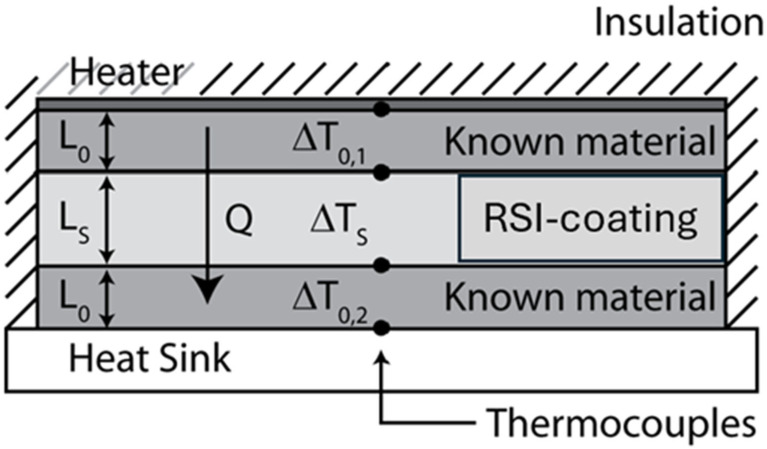
Heat flow meter setup for thermal conductivity measurement.

We used a heat flow meter setup based on [16] to measure the thermal conductivity of low-conductivity materials (≤0.15 W/mK). The heat flow meter (Figure 5) was based on one-dimensional, steady conduction through a stack of materials. A sample of unknown thermal conductivity was placed between two pieces of reference material with known thermal conductivity. A heater (Omega Engineering, Norwalk, CT, USA) was placed on top of the upper reference material, and the lower reference material was in contact with a heat sink (Thermo Fisher Scientific, Waltham, MA, USA). Four thermocouples (Omega Engineering, Norwalk, CT, USA) were installed at the interfaces between materials. The heater was turned on at the start of an experiment, and the temperature readings were monitored to determine when steady-state behavior was achieved. The slopes of the temperature readings moved toward zero as equilibrium was reached. The primary outputs of the experiment were the temperature differences across each material. The heat transfer rate through the sample was inferred from the temperature differences across the reference materials.

Conduction through the materials was modeled with Fourier’s law, as shown in Equation (1), and the heat transfer rate through all three materials was assumed to be the same (1D conduction with no heat loss).

(1) $$\overset{•}{Q}=k_{\mathrm{ref}}A\frac{\Delta T_{0.1}}{L_{0}}=k_{\mathrm{RSI}\_\mathrm{coating}}A\frac{\Delta T_{s}}{L_{s}}=k_{\mathrm{ref}}A\frac{\Delta T_{0.2}}{L_{0}}$$

The rate of heat transfer ($\overset{•}{Q}$) was equal to the thermal conductivity for the PVC plates ($k_{\mathrm{ref}}$) of 0.10 W/mK multiplied by the cross-sectional area (A) (temperature drop $\Delta T_{0.1}$ divided by the initial thickness ($L_{0}$).

Some heat was lost from the lateral faces of the stack. Thus, the heat transfer rate through the sample was between the values determined from the upper and lower reference. Therefore, we used the average temperature differences measured for the two references. Given that the cross-sectional area was identical for all three materials, we determined the sample thermal conductivity from the reference material’s thermal conductivity, the measured temperature differences, and the thicknesses of the three materials, according to Equation (2):

(2) $$k_{\mathrm{RSI}\_\mathrm{coating}}=k_{\mathrm{ref}}\frac{\Delta T_{0,avg}}{\Delta T_{s}}\frac{L_{s}}{L_{0}},$$

where $\Delta T_{0,avg}$ is the average of $\Delta T_{0.1}$ and $\Delta T_{0.2}$. In Equation (2), the heat flux through the sample is assumed to be the same as the average of the heat flux through the two reference material sections.

#### 2.3.3. Thermal Decomposition and Char Formation

The thermal degradation behavior of RSI coatings was investigated using TGA (TA Instruments, New Castle, DE, USA) to assess mass loss characteristics and thermal stability. The analysis revealed four distinct thermal decomposition phases: softening (~200 °C), significant mass loss and gas expansion (250–300 °C), char formation (~400 °C), and gradual char degradation (~550 °C), with overlapping transitions clarified through dTGA peaks.

The analysis was conducted under an inert nitrogen atmosphere to prevent oxidative degradation and isolate the pyrolytic decomposition mechanisms. The TGA and derivative thermogravimetry curves provided insight into the RSI coating system’s thermal stability, decomposition kinetics, and char yield. The experiments were performed over a temperature range of 50 to 800 °C under high-purity laboratory-grade nitrogen at a constant flow rate of 50 mL/min to ensure an oxygen-free environment. A uniform heating rate of 10 °C/min was applied across all tests to achieve a controlled thermal degradation profile, thereby facilitating accurate interpretation of thermal events, decomposition phases, and residue formation critical to the fire-retardant efficiency of the RSI coatings.

### 2.4. SEM and MATLAB Analysis of Char Morphology and Porosity

To further examine the influence of heat treatment on the intumescent char’s porosity and structure, we assessed how char layers were sectioned from sample centers and adhered to specimen holders with conductive adhesive for SEM imaging at 100× magnification with a 20 kV excitation voltage (Figure 4e).

The SEM imaging process captured detailed microstructural features at high magnification and enabled the identification of voids (black regions) and solid matrices (white regions).

The images were processed in MATLAB (MathWorks, Natick, MA, USA, R2021a) according to established methods from Athi Narayanan’s K-means-based color palette reducer and [32]. Ten SEM images were analyzed per sample, with a reproducibility error of ±3% based on triplicate measurements.

Depth mapping, binary segmentation, and pore space segmentation were used to quantify porosity changes after heat exposure. MATLAB-based image processing techniques assigning grayscale values (0–255) to individual pixels enabled precise porosity measurements through depth map calculations and binary segmentation. This integrated approach provided a comprehensive understanding of how porosity influences thermal resistance by analyzing the void distribution, pore connectivity, and matrix integrity, which collectively determine the heat dissipation, insulation efficiency, and expansion in RSI coatings. The findings offered crucial insights into heat transfer behavior and fire protection performance by correlating microstructural changes with thermal conductivity, expansion kinetics, and char stability under fire exposure.

The combined SEM and MATLAB analysis provided a detailed assessment of porosity evolution in RSI coatings under thermal exposure and revealed a dynamic interplay among gas release, char densification, and decomposition (Figure 4d). In the first 15 min, the porosity increased because of volatile gas release from decomposing APP and TA, thus initiating intumescence and pore formation. At 30 min, the porosity declined as the foam collapsed and char densification occurred, thereby stabilizing the structure. After 60 min, the porosity increased again because of secondary expansion from residual decomposition, and the formation of additional voids and microchannels enhanced the thermal insulation. These structural transformations influenced heat resistance and mechanical integrity: early-stage porosity improved thermal insulation, whereas later-stage char densification strengthened coating adhesion. This integrated approach offers critical insights for optimizing RSI coating performance by balancing expansion, porosity control, and fire resistance efficiency.

## 3. Results and Discussion

### 3.1. Thermal Decomposition and Char Formation

**Figure 6:**
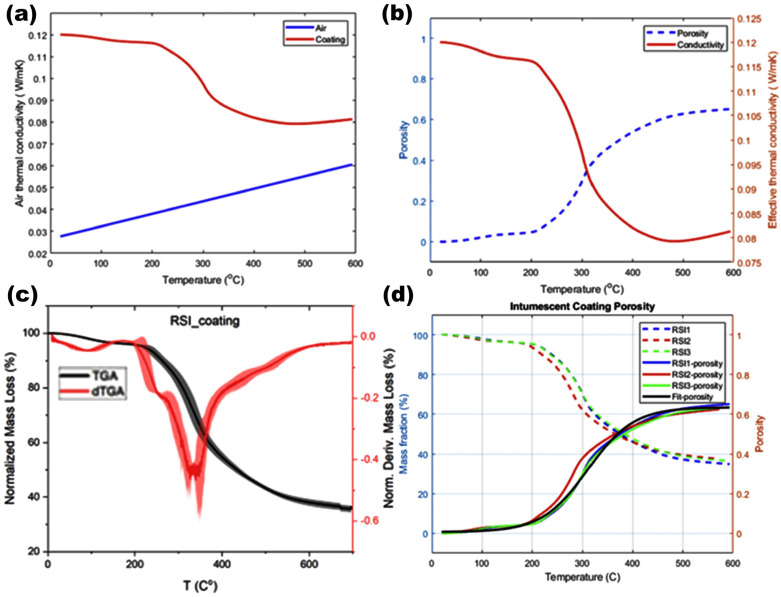
(**a**) Air and coating thermal conductivity in intumescent coatings across temperature range; (**b**) evaluating temperature-induced changes in porosity and thermal conductivity of intumescent coatings; (**c**) mass loss and rate of mass loss (dTGA) of the RSI coating; (**d**) influence of fire exposure on porosity development and mass reduction in RSI coatings with curve fitting. Note: the maximum value of porosity in Equation (7) is 62%.

The TGA and dTGA curves in Figure 6c illustrate the thermal degradation profile of the RSI coating and highlight the decomposition stages. Compared with other ICs [8], the RSI coatings exhibited lower expansion (1.2%) but stronger adhesion—suggestive of a more compact and stable char structure—thus outperforming commercial coatings under light agitation, with a direct comparison planned for future work.

The black TGA curve, representing the normalized mass loss (%), shows an initial gradual decomposition up to 200 °C, which was followed by a sharp mass loss between 250 °C and 400 °C indicating intense pyrolysis and char formation. The red dTGA curve shows the degradation rate, which peaked between 350 °C and 400 °C, corresponding to the breakdown of flame-retardant additives (APP and TA) and the expansion of the intumescent structure. Beyond 400 °C, the stabilization of mass loss indicated the development of a thermally stable char layer enhancing heat insulation and fire resistance. The analysis revealed four distinct thermal decomposition phases: softening (~200 °C), significant mass loss and gas expansion (250–300 °C), char formation (~400 °C), and gradual char degradation (~550 °C). These findings confirmed the RSI coating’s effectiveness in forming a protective insulating barrier under elevated temperatures.

Building on the thermal decomposition insights obtained from TGA, we further quantified the porosity evolution in the RSI coating with MATLAB-based image processing techniques. As the material decomposed and underwent phase transitions, the formation of voids and microstructural changes directly correlated with the mass loss profile captured in TGA. To systematically calculate porosity (ϕ), we used Equation (3):

(3) $$\phi =1-\left(\frac{\omega }{100}\right),$$

where (ω) denotes the normalized mass fraction obtained from TGA data, and 100 is the percentage.

To further analyze the influence of chemical composition on porosity evolution, we formulated three variations in RSI coatings—RSI1, RSI2, and RSI3—with slight modifications in the percentages of key intumescent additives, such as APP and TA. These variations were designed to systematically investigate the effects of compositional adjustments on thermal decomposition, mass loss, and porosity development under increasing temperatures.

In Figure 6d, the mass fraction curves (dashed lines) represent the thermal degradation profiles of RSI1, RSI2, and RSI3. In contrast, the corresponding porosity curves (solid lines) illustrate the expansion behavior and void formation as a function of temperature. The primary objective of this comparison was to evaluate how small compositional changes influence char structure stability, expansion kinetics, and overall fire protection performance.

A fitted porosity curve (black solid line) was added to enhance the predictive model’s accuracy through curve-fitting techniques. This fit provides a smoothed representation of porosity evolution, thereby decreasing experimental noise and ensuring a more reliable correlation between mass loss and expansion. By incorporating this fitted curve, we sought to establish a generalized trend that may be used in predictive modeling for optimizing RSI coatings. This comparative approach deepened understanding of how specific formulation adjustments affect the porosity-thermal conductivity relationship, thus providing valuable insights into the design of next-generation fire-retardant coatings with improved performance and stability.

### 3.2. Temperature-Dependent Porosity

After quantifying porosity from TGA-derived mass loss data with Equation (3), we further analyzed its temperature-dependent behavior to establish a more predictive model. To establish a temperature-dependent porosity model, we quantified the porosity (*ϕ*) with TGA-derived mass loss data and conducted further analysis with MATLAB-based computational techniques. The porosity function is defined as Equation (4):

(4) $$\phi (T)=C_{4}+C_{3}*\tanh(C_{2}*(T-C_{1})),$$

where

$C_{1}$ represents the temperature shift (onset of significant porosity change),
$C_{2}$ controls the spread/steepness of the transition or the curve, dictating how quickly porosity changes with temperature,
$C_{3}$ is the amplitude (maximum change in porosity), and
$C_{4}$ is the offset (porosity at very high temperatures).

When Equation (4) was used to fit the data in Figure 6d with MATLAB, the coefficients were found with Equation (5):

(5) ϕ(T)=0.3210+0.3142∗tanh(0.011∗(T−310.6)).

This function characterizes the evolution of porosity as a function of temperature and accurately captures the sigmoidal trend observed during thermal decomposition. The porosity initially remained low at lower temperatures and was followed by a rapid increase beyond 300 °C, marking the onset of char expansion with predominantly closed-cell porosity (confirmed by SEM, Figure 4e). This structure enhanced insulation by trapping gas, reducing conductivity to 0.05 W/mK at 62% porosity. However, the potential for excessive porosity to compromise mechanical stability [16] may represent a trade-off to be explored in future adhesion testing.

The applied curve-fitting technique aligned with the methods of W.A.M. Aarnink et al. with a fit error of ±5%, thereby ensuring a precise representation of porosity evolution. Integrating these fitted porosity curves into a predictive model enhanced the correlations among porosity dynamics, heat transfer behavior, and fire protection performance, and provided critical insights into IC efficiency under thermal exposure.

While the RSI coating evaluated in this study exhibited negligible initial porosity due to its formulation and uniform application, the porosity function derived in Equation (4) is adaptable to coatings with non-zero initial porosity. This is achieved by adjusting the offset parameter (*ϕ*_0_), which sets the baseline porosity value prior to thermal exposure. In real-world applications, many coatings may contain inherent porosity from application methods, solvent evaporation, or curing inconsistencies. Our model accommodates this by shifting the initial point of the porosity–temperature curve upward, thus reflecting the presence of such voids. This modification preserves the model’s accuracy in predicting the thermal behavior of coatings with variable initial porosity and reinforces its broader applicability in practical fire protection engineering.

### 3.3. Thermal Conductivity

To accurately determine the thermal conductivity of the RSI coating (i.e., the thermal conductivity of the coating matrix), we used a heat flow meter setup according to a method described by [16]. The setup relied on a 1D steady-state conduction model in which the RSI coating sample was sandwiched between two reference materials with known thermal conductivity. A controlled heat source (electrical resistance heater) was applied to the upper reference material to provide consistent thermal input, and the lower reference material was placed in direct contact with a metallic heat sink at ambient temperature. This configuration ensured a stable, unidirectional heat flow and a steady-state thermal gradient across the test stack, which is essential for applying Fourier’s law to accurately measure thermal conductivity.

Thermocouples at each interface continuously recorded temperature differences and enabled precise monitoring of equilibrium conditions. The heat transfer rate through the stack was governed by Fourier’s law, in which the steady-state heat flux was assumed to be constant across all layers. The thermal conductivity of the RSI coating was calculated with Equation (2), considering the measured temperature gradients, material thicknesses, and known conductivity of the reference layers. To account for lateral heat losses, we used the average temperature differences from both reference materials to estimate the effective thermal conductivity.

The results demonstrated that the RSI matrix exhibited a thermal conductivity of 0.12 W/m·K ± 0.006 W/m·K (±5%) based on triplicate measurements, thus highlighting its low heat transfer characteristics and strong insulation performance. This value, K_matrix_, integrated the effects of solid-phase conductivity and gas-phase insulation within the expanded coating contributing to the coating’s fire protection efficiency. The thermal conductivity of air was incorporated with [27] linear equation, which represents the gas in the pores of the expanded material. [27] linear equation, as given in Equation (6),

(6) $$K_{air}=0.0109+5.74\times 10^{-5}T,$$

ensured consistency with the experimental setup. To develop a comprehensive representation of heat transfer within the expanded RSI coating, we applied the following porosity-based thermal conductivity equation:

(7) $$K_{RSI\_coating}=\left(1-\phi \right)K_{matrix}+\phi K_{air}.$$

The resulting temperature-dependent thermal conductivity curve (Figure 6a) was generated with the mixture model in Equation (7). The combined model effectively captured the interplay between solid-phase and gas-phase contributions, offering a refined predictive tool for assessing the RSI coating’s thermal insulation performance under high-temperature fire scenarios.

The thermal conductivity behavior of the RSI coating was closely associated with its porosity evolution during thermal decomposition. The effective thermal conductivity curve was derived by integrating experimental measurements with theoretical modeling, incorporating the virgin RSI thermal conductivity at room temperature and the porosity fraction obtained from mass loss analysis (Figure 6b). This combined approach comprehensively evaluated how gas-phase contributions and solid-phase structural changes influenced heat transfer within the coating.

An inverse relationship between porosity and effective thermal conductivity was observed with increasing temperature. As the porosity increased, the effective thermal conductivity sharply decreased to approximately 300 °C and from 0.15 to 0.08 W/m·K. These findings suggested that structural changes in the coating created a porous char layer, thus enhancing thermal insulation capability. At approximately 400 °C, the porosity stabilized and the thermal conductivity plateaued; these findings indicated the formation of a stable char structure acting as an effective thermal barrier. This consistent decrease in thermal conductivity at higher temperatures underscores the importance of controlled porosity in enhancing the thermal resistance of fire-retardant coatings and indicates their suitability for high-temperature applications requiring robust fire protection. Findings from I.H. Tavman [24] support the results presented herein, thus reinforcing our data’s reliability.

### 3.4. Numerical Prediction of Thermal Performance in RSI Coatings with COMSOL Multiphysics

To overcome the challenge of experimentally measuring the backside temperature profiles of RSI coatings during fire exposure, we used COMSOL Multiphysics to simulate the heat transfer process in test 2 in the experiment. In this test, the coated material was placed in an oven for more than 20 min. The specimen was initially maintained at 20 °C and placed in the heating chamber, which was maintained at 400 °C, the critical threshold for structural steel integrity, thus ensuring standardized boundary conditions for evaluating insulation performance.

The modeling of heat transfer across the RSI coating incorporated key material properties, including porosity, density, air thermal conductivity, thermal expansion, and coating-specific characteristics, all derived from experimental data. By integrating both solid-phase and gas-phase contributions, the material properties used in COMSOL Multiphysics enabled accurate prediction of heat transfer dynamics (Table 2) and provided critical insights into the time-dependent evolution of the backside temperature profile and the RSI coating’s fire protection efficiency.

The predicted backside temperature profile of the RSI coating over a 20 min exposure period, building upon the numerical modeling framework, is illustrated in Figure 4c. The COMSOL Multiphysics simulation effectively captured the heat transfer dynamics and highlighted the difference between the surface (exposed) and backside (protected) temperatures. The surface temperature rapidly stabilized at 400 °C, aligning with the fixed radiative temperature in the heating chamber, whereas the backside temperature gradually increased before reaching equilibrium. This delayed heat penetration was attributed to the formation of an expanded intumescent char layer, which enhanced insulation by impeding conductive and convective heat flow. The model validated the thermal protective efficiency of the RSI coating, with an average deviation of <5% from experimental data, thus demonstrating its ability to maintain a lower backside temperature and consequently prevent the underlying steel substrate from reaching critical failure thresholds. A sensitivity study on porosity (varied ±10%) showed a 3–5% impact on predicted temperatures, reinforcing model robustness.

## 4. Conclusions

This study systematically evaluated RSI coatings’ thermal resistance and porosity evolution under fire exposure and demonstrated their effectiveness in insulating metal substrates. Our experimental results confirmed that the formation of a porous char layer significantly decreased heat transfer: the porosity reached 45% after 60 min of exposure and led to a thermal conductivity decrease from 0.15 W/mK to 0.05 W/mK. SEM and image processing analyses validated the char’s structural integrity, and numerical heat transfer modeling further supported the inverse correlation between porosity and conductivity. The RSI coating exhibited strong thermal stability and expansion and was, therefore, considered suitable for high-performance passive fire protection. This behavior was confirmed by experiments using fire exposure, SEM imaging, and heat flow tests, along with COMSOL simulations. This study demonstrates that controlled porosity evolution—quantified via SEM and MATLAB analysis—directly reduces effective thermal conductivity and enhances the fire protection performance of RSI coatings. These findings offer a predictive framework for designing bio-derived intumescent coatings with tailored pore structures optimized for thermal insulation in high-risk industrial settings. The RSI formulation, with its thin-film application, strong adhesion, and decreased emissions, meets current industry needs for performance, safety, and sustainability, and therefore may be a promising choice for future fire protection in the oil and gas industry.

## Figures and Tables

**Table 1:** Formulation and coating composition.

System	Component	Content (%)
**RSI**	TA	28
	APP	6.8
	EPOXY	65
	Polyamine	21.2

**Table 2:** Material properties and parameters.

*Material Property*	*Value*	*Comments*
*Thermal Conductivity of K_matrix*	0.12 W/km	In this work
*Specific Heat Matrix* *CP_Matrix*	1500 [J/kg/K]	In this work
*Specific Heat* Cp_air	$55.5*(a_{1}+a_{2}*T+a_{3}*T^{2}+a_{4}*T^{3})$	$a_{1}$ = 971, $a_{2}$ = 0.06, $a_{3}$ = 1.66 × 10^−4^, $a_{4}$ = −6.79 × 10^−8^
*Air thermal conductivity* *K_air*	$1*\left(0.0109+5.74e-5*T\right)$	
*Thermal expansion*	alpha*(1-p1(T))	Alpha = 1.0 × 10^−2^ (mm/s/k) p1 = the value from Equation (4)
*Porosity (* ϕ(T) *)*	From Equation (4)	From porosity values

## Data Availability

The original contributions presented in this study are included in the article. For further inquiries, please contact the corresponding authors.
